# Microbial contamination in the indoor environment of Arabian Gulf: a review

**DOI:** 10.3389/fmicb.2026.1796861

**Published:** 2026-03-31

**Authors:** Gabriel Haddad, Mohamad Al Hallak

**Affiliations:** 1 American University of the Middle East, Egaila, Kuwait; 2 College of Engineering and Technology, American University of the Middle East, Egaila, Kuwait

**Keywords:** bacteria, fungi, GCC countries, health risks, indoor air quality, sampling

## Abstract

This review investigates indoor microbial contamination across Gulf Cooperation Council (GCC) countries (Saudi Arabia, the United Arab Emirates, Qatar, Kuwait, Bahrain, and Oman) over the last decade. Investigations on indoor fungal and bacterial contamination took place in different building types (residential, commercial, educational, and healthcare buildings). Across the studies, recurrent fungal genera (*Aspergillus, Penicillium, Cladosporium, Alternaria, and Fusarium*) and common bacterial genera (*Staphylococcus, Bacillus, and Micrococcus*) were frequently reported in various samples as airborne bioaerosols, on damp building materials, on heating, ventilation and air conditioning (HVAC) components, and in settled dust. These loads are often exacerbated by regional heat and humidity conditions, dust intrusion, and heavy reliance on air conditioning. Reported concentration ranges of microbial contaminants are accompanied by health-related risk assessments for the most identified microbial taxa indoors. Significant inadequacies are noted in this review, including inconsistent country coverage, a shortage of studies on indoor biological contaminants, and a lack of comparable air, surface, and dust sampling. The need for regional recommendations, standardized sampling and reporting procedures, moisture and condensation control, strict HVAC cleanliness and filtration, the deterioration of indoor air quality, the danger of infectious exposure and regular monitoring for direct remediation are highlighted here. These actions can reduce microbial loads and improve indoor environmental sustainability across the region.

## 1 Introduction

While people spend almost all of their time indoors in places ranging from houses, companies, schools, hospitals, and gathering places, indoor environments are now recognized as the primary setting for human exposure to biological contaminants (Mannan and Al-Ghamdi, 2021). Fungi and bacteria are among these agents that influence air quality by their spores, cells, fragments, toxins, and microbial volatile organic compounds (World Health Organization, 2009; World Health Organization. Regional Office for Europe, 2010). Moisture, temperature, ventilation, occupant activity, and the bio-receptivity of materials all have a significant impact on their occurrence and persistence (Al Hallak et al., 2023; Brambilla and Sangiorgio, 2020; Borrego and Molina, 2019; Ellringer et al., 2000). Though these factors are present everywhere, the Gulf Cooperation Council (GCC) region offers a unique set of conditions, including arid weather, intense heat, seasonal humidity, saline/coastal air masses, frequent dust storms, and a year-round reliance on mechanically cooled buildings (Patlakas et al., 2019). Microbial reservoirs and resuspension indoors can be amplified by these conditions, as they promote episodic surface condensation, quick dust deposition in textiles and heating, ventilation and air conditioning (HVAC) components, and significant swings in indoor–outdoor pressure and airflow. Based on ventilation and maintenance modes, indoor moisture content may reach a level favorable for microbial growth and thus human exposure. Indeed, high-occupancy indoor buildings (mosques, classrooms in schools, universities, etc.) usually have short and dense occupancy which may affect ventilation and indoor air quality (IAQ) dynamics if not well controlled (Al-Homoud, 2009; Al-Anazi and Almasri, 2023).

Monitoring IAQ and HVAC hygiene is thus important within all indoor settings but are also critical in the healthcare estate (Hospitals, Healthcare centers, Clinics, etc.) which is expanding rapidly in the region (Amoatey et al., 2018). In response to these factors, local authorities in the GCC region underlined, unevenly, guidelines for IAQ control considering ventilation design standards and ambient-air frameworks with limited pollutant-specific indoor standards underlined and thus few or no recommendations about specific indoor biological contaminants (Amoatey et al., 2018; Liu, 2021). The aim of this study is to investigate the current situation of indoor biological contaminants in the GCC region, highlight their potential consequences on the occupants’ health and to underline the importance of monitoring IAQ while considering the risk of exposure to these contaminants. Although airborne microbial communities are present all around and have been widely investigated worldwide, a limited number of studies have explored these microbes in indoor settings in the GCC countries over the last decade. Airborne contaminants could originate from a variety of natural sources, most notably soil, water, and dust in the atmosphere. Especially in arid areas such as the GCC countries, where frequent dust storms can carry airborne pathogens. These are subsequently mobilized into urban areas, and frequently concentrated within indoor environments, particularly through air conditioning (AC) systems. Although indoor air is extensively researched worldwide, there is not an in-depth assessment of data specific to the built environment of the GCC, which results in inconsistent standards and delays the establishment of region-specific regulations. In fact, the area has seen rapid improvements in ventilation techniques, high-efficiency HVAC systems, green-building regulations, and building materials during the last 10 years, all of which have a significant impact on indoor contaminants. In this review, we present investigations performed in the last decade (2015-2025) with a focus on indoor fungal and bacterial contamination in the GCC region in different building types. Focusing on the last decade enabled a more coherent synthesis, ensuring the findings remain directly relevant to current indoor air policy development in the region. Sample types (airborne, surface, dust), sampling methods, analysis methods, fungal and bacterial genera/species, and quantification of identified taxa (when available) were summarized. In addition, the sampling strategy followed in each study including information of how samples were collected, is there paired indoor-outdoor sampling, spatial coverage across rooms/floors, and/or temporal coverage across seasons, etc. Although few studies are available in the last decade, most identified taxa are highlighted with their possible consequences on human health upon exposure. Additionally, key points for monitoring IAQ in the GCC region and priorities for future research and policy development are emphasized (Habibi et al., 2021; Habibi et al., 2022).

## 2 Methodology

This review was conducted following a structured literature search approach limited to the Gulf Cooperation Council (GCC) countries (Saudi Arabia, United Arab Emirates, Qatar, Kuwait, Bahrain, and Oman). Peer-reviewed articles published in the 2015-2025 decade were targeted through different research databases (PubMed, Scopus, Web of Science). The initial search included a combination of terms related to indoor air quality, fungal and bacterial contamination, surface, airborne, dust, bioaerosols, in addition to the names of countries mentioned above.

Then, the studies included in this review were selected from the initial search based on the following criteria: (i) Studies conducted in one of the GCC countries in the last 10 years; (ii) investigations took place in indoor environments (residential, educational, healthcare, commercial, industrial, or religious buildings); (iii) studies reported original microbial sampling campaign results; (iv) investigations targeted either fungal or bacterial contamination or both. Studies that only did outdoor sampling, targeted non-microbial pollutants, or were conducted before the defined time frame were excluded.

## 3 Bacterial contamination in buildings

Bacterial contaminants indoors come from a variety of sources which are typically environmental or human activity related. Environmental species such as *Bacillus* and *Streptomyces* as well as other bacteria considered dust-borne were detected by several studies conducted on indoor air. In contrast, indoor air could also carry various bacteria from human sources, often bacteria coming from normal flora such as *Staphylococcus* and *Micrococcus*, among many others. Some pathogenic bacteria would also be concentrated in an indoor setting, especially in buildings such as hospitals and research facilities involved with diagnosing patients (Habibi et al., 2023; Abdelrahman et al., 2022; Almatawah et al., 2022; Jung et al., 2024). Moreover, indoors, the central AC systems concentrate and disperse microorganisms throughout significant areas of the buildings. Re-suspended coarse particles, which may contain biological contaminants, are captured by AC filters, offering a snapshot of the cumulative exposure to these bio-contaminants. Several studies have investigated these filters to explore the indoor air microbiome (Viegas et al., 2014; Wylick et al., 2021; Seo et al., 2008).

Bacteria detected and identified in indoor settings in Kuwait, KSA, Qatar, UAE and Oman were grouped in Table 1. The data was collected from all published papers available in the literature between 2015 and 2025.

**Table 1:** A summary of indoor investigations on bacterial contamination on surfaces, airborne and from dust in the GCC within the period of 2015-2025.

Country and study	Kuwait (Habibi et al., 2021)	Kuwait (Habibi et al., 2022)	Kuwait (Habibi et al., 2023)	Kuwait (Almatawah et al., 2022)	Qatar (Abdelrahman et al., 2022)	UAE (Jung et al., 2024)	KSA (Basha Sheik, 2015)	KSA (29)	KSA (Al-Abdalall and Al-Abkari, 2017)	KSA (AlMulla et al., 2022)
Sampling	Airborne							Dust		
Building type	H	H	H	W	H	Ed	Ed	R-Ed	R	R-W
Year	2021	2022	2023	2022	2022	2024	2015	2016	2017	2022
Identification										
Method (culture/molecular)	molecular	molecular	molecular	culture	culture	culture	culture	culture	culture	culture
*Gram negative*	+			+	+			+	+	+
*Gram positive*	+			+	+		+	+	+	+
*Acinetobacter* spp.*				+	+					
Arthrobacter*					+					
Arthrospira*		+								
*Bacillus* spp.*				+	+		+	+	+	+
Comamonas*		+								
Corynebacterium*					+					
Haemophilus influenzae*	+									
Methylibium**		+								
Micrococcus*					+		+			
Mycoplasma pneumoniae*	+									
Oligella ureolytica*									+	
Parvibaculum**		+								
Pseudomonas*					+					
Pseudonocardia**		+								
Roseomonas*					+					
*Serratia* spp.*									+	
Sphingobium*		+								
Sphingopyxis**		+								
*Staphylococcus* spp.*				+	+		+	+	+	+
Stenotrophomonas*				+	+					
*Streptococcus* spp.*	+									
Taonella**		+								
Variovorax*		+								
Zoogloea**		+								

+, Bacteria detected or isolated; ^*^, clinically relevant bacteria; ^**^, environmental bacteria; Ed, educational buildings (schools, universities); H, health care centers (hospitals); R, residential (houses, dwellings in buildings); W, workplaces (offices, industrial, agricultural zones).

Investigations on the presence of bacteria were held in hospitals and healthcare centers (Habibi et al., 2021; Habibi et al., 2022; Habibi et al., 2023; Abdelrahman et al., 2022), educational buildings (Almatawah et al., 2022; Jung et al., 2024; Basha Sheik, 2015), residential, industrial, and agricultural buildings (AlMulla et al., 2022). Bacterial detection was performed on airborne and dust samples. No studies on surface samples were performed during the last decade. The analyses were based on culture methods for living bacterial cells, or molecular methods including PCR and Real time PCR for living and dead bacteria. Identification of the bacterial species in the analyzed studies was performed using traditional methods such as colony identification or Gram staining, and molecular methods including 16S rRNA sequencing or New Generation Sequencing and metagenomics. Qualification of the relevant bacterial species was based on colony forming (CFU) or metagenomic analyses and relative abundances.

### 3.1 Airborne bacterial contamination

A total of seven papers over the last decade investigated airborne bacterial presence in indoor aerosols of GCC countries. Across three studies in Kuwaiti hospitals, several strategies were used to investigate the indoor aerosols compared to outdoors, such as detecting specific bacterial pathogens that could be problematic, investigating the overall bacterial communities, or identifying antibiotic resistance genes (ARGs) in hospital environments and highlighting their potential risks in such settings (Habibi et al., 2021; Habibi et al., 2022; Habibi et al., 2023). A first study analyzed aerosols during the COVID-19 period, investigating the presence of airborne pathogenic bacteria, alongside common respiratory viruses indoors in three hospitals and a research facility actively involved in coronavirus testing (Habibi et al., 2021). All four facilities investigated had a strong human presence, especially infected patients and people at risk of infection, despite the measures taken to limit the spread of the virus. Interestingly, *Haemophilus influenzae* was only detected indoors in hospitals, and not in the research facility. Moreover, in one of the three hospitals, two other bacterial pathogens were detected airborne: *Streptococcus pneumoniae* and *Mycoplasma pneumoniae*. The study highlighted the risk of these bacterial pathogens when associated with a SARS-CoV-2 infection, specifically in the COVID-positive areas of the hospitals. In another study, the same team investigated bacterial communities in two of the abovementioned hospitals and the research facility (Habibi et al., 2022). The researchers investigated the bacterial communities present, analyzed the intra-site variability, and compared hospital to non-hospital settings. The viable bacterial load and specific pathogens were targeted in a culture-independent method. Proteobacteria phylum was dominant among bacteria, and the authors reported the presence of several genera previously described as opportunistic human pathogens, such as *Variovorax, Sphingobium, Comamonas,* and *Arthrospira*. Other environmental bacterial genera were also detected indoors (*Parvibaculum, Pseudonocardia, Taonella, Methylibium, Sphingopyxis,* and *Zoogloea*). Additionally, a very low relative abundance was recorded for the ESKAPE pathogens (*Enterococcus faecium, Staphylococcus aureus, Klebsiella pneumoniae, Acinetobacter baumannii, Pseudomonas aeruginosa,* and *Enterobacter* species). The bacterial composition was variable in hospital and non-hospital settings, while the fungal community was more homogeneous, even compared to outdoor environment. Remarkably, the abundance and richness of species were greater in ambient air samples compared to indoor samples from both hospital and non-hospital environments. This was probably due to the rigorous hygiene and disinfection protocols implemented in hospitals as a result of the SARS-CoV-2 outbreak, a phenomenon extensively described in the literature (Peters et al., 2022). The authors highlighted the importance of investigating the resistome in aerosols, which they investigated in a third study (Habibi et al., 2023). In the latter, air samples from the same collection sites were analyzed over several months, covering the fall and winter seasons in Kuwait. The total bacterial load was higher in the winter season, suggesting that during winter pathogenic bacteria were more prone to proliferation. Evidently, the total bacterial load was more important indoors (10^4^–10^5^/m^3^) compared to outdoor air (10^2^/m^3^). More importantly, in this study, the authors investigated the presence of ARGs of clinically relevant bacteria. A total of 52 ARGs were positively detected indoors and outdoors, with a slightly higher diversity indoors, most likely due to higher human presence in enclosed areas. Winter season had a more significant richness and evenness of ARGs in aerosols, and the human footfall had major impact on the resistome of the different sites. Alarming results were found when characterizing the ARGs, where beta-lactams (class A, B, C and D) were the most dominant class, followed by ARGs coding for Fluoroquinolones, multidrug resistance (MDR), aminoglycoside, erythromycin, and macrolide-lincosamide-streptogramin B (MLSB). Fortunately, vancomycin and sulfonamide ARGs were only detected in one of the three hospitals. Beta-lactams are considered as a last-resort drug for serious and severe bacterial infections, and the most concerning of those were the imipenemase (*IMP-2 group*) detected at a prevalence of 85%. Following these results, authors underlined the risk of these ARGs carried within the general population, which pose a threat of high possibility of nosocomial outbreaks, as well as a significant risk factor for healthcare professionals and patients. They concluded by highlighting the importance of regular ARG monitoring, hospitals being a hotspot for transmission of such resistant microbes.

In another study, a 19-story complex building with open-space offices was also assessed in Kuwait using a six-stage Anderson air sampler (Almatawah et al., 2022). The samples collected indoors and outdoors were processed through culture methods for quantification (CFU/m^3^), and the most frequent bacterial colonies were identified using a metabolic fingerprinting biochemical analysis. The total bacterial concentration ranged between very low (35 CFU/m^3^) and very high (18,463 CFU/m^3^). A common trend was observed as these values varied with the seasons, significantly increasing in the fall and winter seasons, with a tendency of increased contamination levels in the morning, for most of the analyzed floors. *Bacillus pseudomycoides*/*cereus* (75.7%) were the most prevalent isolates, followed by *Acinetobacter nosocomialis* (11.7%), *Staphylococcus haemolyticus* (3.6%), and *Stenotrophomonas maltophilia* (1.9%). Species found normally on human skin were also identified. Some of the identified species (*Bacillus pseudomycoides*/*cereus*) are typically found in environmental samples, while others are in fact opportunistic pathogens. The indoor air had a higher bacterial count than the outdoor air in winter, spring, and summer. The authors concluded the study with a high – extremely high – bacterial contamination level, yet with no indication of any potential risk for the health of building occupants, since pathogenic species were of low frequency.

On the other hand, the work of Abdelrahman et al. (2022) assessed the air quality in different areas (lobby, triage, and outdoor) of four healthcare centers in Qatar during the COVID-19 pandemic. The researchers used a six-stage cascade impactor to analyze the presence of airborne culturable bacteria in the inhalable and the respirable stages, simulating the upper and lower airways. To investigate culturable bacteria, the analysis was based on culture methods, namely colony forming units per m^3^ (CFU/m^3^) for the quantification of the total bacterial count and a selective culture media for the specific detection of *Methicillin-Resistant Staphylococcus aureus* (MRSA) with further identification using Gram staining. In addition, a 16S rRNA PCR and HISeq sequencing analysis was done on the same samples for a specific identification of bacterial isolates and a biodiversity analysis. The total bacterial load surpassed the WHO guidelines (300 CFU/m^3^) in 46.4% of the air samples (3 out of 4 sites), exhibiting a higher viable bacterial concentration inside at these centers. There were no significant differences between lobby and triage areas. The highest colony count was detected in the 1.1-2.1 μm stage of the impactor, simulating the human alveoli and bronchi in the lower airways, which can pose serious risks of nosocomial infections especially in immunocompromised patients. Regarding the molecular analysis, the authors detected several opportunistic bacteria, most abundantly *Staphylococcus*, followed by *Acinetobacter*, *Pseudomonas* and *Bacillus*, among others. They noticed a change in the relative abundances of these bacteria depending on the season, recording a higher alpha-diversity and an increase of *Staphylococcus* during the cold season.

In United Arab Emirates, a study analyzed the IAQ of some schools in Dubai, assessing a series of factors, among which was the airborne bacterial count (Jung et al., 2024). A total of 500 L of air was collected from various rooms (classrooms, labs, computer labs) using an air sampler. Bacterial concentration was measured, yielding concentrations that ranged from 202 to 5,256 CFU/m^3^. Surprisingly, the highest concentration was detected in the computer labs, which can be explained by limited ventilation in these areas, as well as a humid climate during the collection season, according to the authors. Moreover, in the classrooms, the authors highlighted an increase in measured values during class sessions. Generally, airborne bacterial concentrations surpassed the required norms and maintenance criteria for IAQ management as defined by the WHO (World Health Organization. Regional Office for Europe, 2010).

In another paper, air samples were collected using the settle-plate method, from a college of medical sciences in Saudi Arabia (Basha Sheik, 2015). Five indoor locations (microbiology laboratory, classrooms, toilets, lift, office rooms) were experimentally analyzed. Bacteria were quantified by CFU, followed by CFU/m^3^ conversion, and were identified using microscopy and biochemical methods after culture. The highest bacterial concentration was recorded in microbiology laboratories (320 CFU/m^3^), while the lowest was in the offices (61 CFU/m^3^). Only Gram-positive bacteria were isolated, with *Staphylococcus* as the most dominant, followed by *Bacillus* and *Micrococcus* species. The research indicated that indoor bacterial concentrations differed by location but consistently fell within the <500 CFU/m^3^ range, which aligns with a low level of contamination as per WHO indoor air guidelines (World Health Organization. Regional Office for Europe, 2010), suggesting that the college environment is microbiologically safe under the surveyed conditions.

### 3.2 Dust bacterial contamination

Regarding the bacterial contaminants in dust samples, studies in the GCC countries over the past decade were limited, as only three investigations were carried out. The first one compared different types of AC filters by collecting dust samples from various buildings in Dammam, KSA (Al-Abdalall and Al-Abkari, 2017). The analysis was realized using culture over the four seasons. The bacterial species isolated were *Serratia liquefaciens, S. lentus, Oligella ureolytica, Bacillus pumilus, B. cereus,* and *B. subtilis.* A higher microbial count was detected in window-type air conditioning units, and the complex AC filters were the most efficient in filtering the air up to 91.8% for bacteria.

In the second paper, AlMulla et al. (2022) collected dust from AC filters from industrial, residential and agricultural buildings. The authors analyzed dust to quantify the bacterial load and characterize the isolated species for their antibiotic sensitivity, hemolytic activity, and Gram stain. The highest bacterial concentration was recorded in agricultural buildings (4.5 × 10^5^ CFU/100 mg of dust), followed by residential buildings (4.5 × 10^4^ CFU/100 mg of dust) and lastly industrial buildings (88 × 10^3^ CFU/100 mg of dust). In all three buildings, Gram-positive were more abundant than Gram-negative bacteria, mainly belonging to *Bacillus* and *Staphylococcus*. Antibiotic susceptibility against four antibiotics was investigated using culture-based methods, and interestingly most of the bacterial isolates from agricultural locations were the most sensitive. Resistance to ampicillin was identified in most bacterial samples of all locations, intermediate resistance to neomycin and kanamycin and showed the most sensitivity to doxycycline. These resistance profiles indicate a potential health risk to human health. The authors also investigated the hemolytic activity of the isolates. Generally, hemolytic activity is considered one of the virulence factors of many bacteria, producing hemolysin toxins and therefore hazardous for human health. A striking 67% of all bacterial isolates from agricultural areas exhibited a potential beta-hemolytic activity, and the remaining 33% showed an alpha-hemolytic activity. In residential buildings, half of the isolates were non-hemolytic, while in industrial buildings 67% had no hemolytic activity. They concluded the paper by highlighting the reliability of analyzing AC filter dust for assessing IAQ and bioaerosols.

In a third study, the dust from AC filters and carpets in homes and several rooms at a university in KSA was investigated (Almoffarreh et al., 2016). The authors used culture to isolate the bacterial biodiversity present in these samples, and biochemical tests to identify them. Mostly *Bacillus* spp. (60.3%) was found, along with *Staphylococcus* spp. (37.9%) and Gram-negative bacilli (1.7%), with a mean bacterial concentration of 1.33 × 10^5^ CFU/g of dust. The investigators concluded with the importance of regular cleaning and safe removal of dust from AC filters and carpets to minimize human exposure to potential bacterial pathogens.

## 4 Fungal contamination in buildings

Fungi are one of the most common contaminants of the indoor environment worldwide, and so in the Arabian Gulf region. As reported by different researchers, fungi have the ability to grow on organic as well as synthetic building materials if enough moisture content is available (Al Hallak et al., 2023; Viegas et al., 2014; Haleem Khan and Mohan Karuppayil, 2012). Most construction materials commonly colonized by fungi include wall papers, wood, gypsum boards, as well as concrete-based materials at high relative humidity (RH) values (Wylick et al., 2021; Al Hallak et al., 2025). Considering possible water exposure from different sources: condensation from air conditioning, roof leaks during infrequent rains, plumbing leaks, involvement of humid outdoor air into cooler indoor areas etc. Fungi colonizing surfaces of building materials will, under suitable conditions, develop, reproduce and, at certain levels of growth, release aerosols (spores and fragments) into indoor air (Seo et al., 2008; Li et al., 2019; Li et al., 2022). These airborne particles are free to circulate in and out of AC systems, spreading contamination. In this section, studies investigating fungal contamination in the indoor environment of different Arabian Gulf countries through surface, airborne, and dust sampling are targeted.

Investigations on fungal contamination from airborne, surface, and dust sampling indoors are condensed in Table 2. The studies included here performed indoor sampling (airborne, surface or dust) in the Arabian Gulf region and were conducted in the last 10 years (2015-2025).

**Table 2:** A summary of indoor investigations on fungal contamination on surfaces, airborne and from dust in the Arabian Gulf region within the period of 2015-2025.

Country and study	Qatar (Rub et al., 2021)	Kuwait (Almatawah et al., 2022)	Kuwait (Habibi et al., 2022)	Kuwait (Asadzadeh et al., 2025)	KSA (Mashat, 2015)	KSA (Najjar, 2025)	KSA (Alsaif et al., 2020)	KSA (Al-Abdalall and Al-Abkari, 2017)	KSA (Hasnain et al., 2017)	KSA (AlMulla et al., 2022)
Building type	Ed	W	H	H	M	Ed	M	R	R	R-W
Sampling	Airborne						Surface	Dust		
Year	2021	2022	2022	2025	2015	2025	2020	2017	2017	2022
Identification										
*Alternaria ^I, A^*	**+**			**+**	**+**	**+**	**+**		**+**	
*Aspergillus ^I, A^*	**+**	**+**	**+**	**+**	**+**	**+**	**+**	**+**	**+**	**+**
*Aureobasidium^A^*							**+**			
*Bipolaris ^I, A^*				**+**			**+**			
*Candida* sp. *^I, A^*							**+**			**+**
*Cladosporium ^I, A^*	**+**	**+**		**+**	**+**	**+**	**+**	**+**	**+**	**+**
*Cryptococcus ^I^*							**+**	**+**		
*Curvularia ^I, A^*	**+**						**+**			
*Drechslera^A^*	**+**									
*Epicoccum^A^*	**+**									
*Eurotium^A^*					**+**					
*Fusarium ^I, A^*		**+**	**+**		**+**	**+**	**+**		**+**	
*Ganoderma^A^*			**+**							
*Heterophoma^E^*			**+**							
*Humicola^E^*			**+**							
*Leptobacillium^E^*			**+**							
*Leptosphaeria^A^*	**+**									
*Malassezia^I, A^*			**+**							
*Mucor ^I, A^*					**+**		**+**		**+**	
*Paecilomyces ^I, A^*				**+**		**+**				
*Penicillium ^I, A^*	**+**	**+**	**+**	**+**	**+**	**+**	**+**		**+**	**+**
*Pleospora^A^*	**+**									
*Rhizoctonia^E^*								**+**		
*Rhizopus ^I, A^*	**+**						**+**		**+**	
*Rhodotorula^I^*		**+**								
*Saccharomyces^I^*			**+**							
*Scopulariopsis^I, A^*							**+**			
*Stachybotrys^A^*										**+**
*Streptomyces^A^*							**+**			
*Trichoderma^A^*						**+**				
*Trichosporun ^I, A^*							**+**			
*Ulocladium ^A^*		**+**					**+**			
*Wilcoxinia^E^*			**+**							

+, Fungus detected or isolated; I, infectious; A, allergenic; E, environmental; Ed, educational buildings (schools, universities); H, health care centers (hospitals); R, residential (houses, dwellings in buildings); W, workplaces (offices, industrial, agricultural zones); M, mosques.

It can be noticed that indoor investigations into fungal contamination are limited in the targeted region. Moreover, most studies cluster in KSA (6 out of 10 studies), followed by Kuwait (3 out of 10) and one study in Qatar. In Oman, Bahrain and UAE, data in the last decade was limited.

Airborne sampling was held either by filtration (Habibi et al., 2022; Mashat, 2015) or impaction (Almatawah et al., 2022; Najjar, 2025; Rub et al., 2021) followed by culture and/or DNA analysis. One surface sampling investigations was found in which surface sampling was done by swabbing followed by fungal culture (Alsaif et al., 2020). Three investigations were found, in which dust was collected by vacuuming and filtration and assessed by culture (AlMulla et al., 2022; Al-Abdalall and Al-Abkari, 2017; Hasnain et al., 2017).

Globally, most common fungal species identified in the indoor environment belong to *Aspergillus, Penicillium, Cladosporium,* and *Stachybotrys* genera (Al Hallak et al., 2023; Belizario et al., 2021). Within the GCC region, although few investigations into fungal contamination have been carried out, *Aspergillus, Penicillium, Cladosporium, Alternaria, and Fusarium* were reported as dominant indoor contaminants (Table 2).

### 4.1 Fungal contamination airborne

Six studies investigated airborne fungal contamination in the indoor environment. Airborne fungal investigations in these studies were conducted in religious, educational, office, and healthcare settings in the GCC region, several consistent patterns emerge. Dominant genera were consistently *Aspergillus, Penicillium, Cladosporium, and Fusarium,* regardless of building type. Notably, these dominant genera have serious health consequences: *Aspergillus* spores have been linked to allergic respiratory disorders and invasive infections for exposed individuals (De Linares et al., 2023); *Penicillium* species are associated with allergic responses and asthma symptoms (Al-Shaarani and Pecoraro, 2024); *Cladosporium* spores are major contributors to allergic respiratory diseases (Hadebe and Brombacher, 2019), and several *Fusarium* species can produce a range of mycotoxins and can cause infections upon exposure (Kokkonen et al., 2010).

Four out of six studies quantified airborne fungal loads indoors, where two of them compare indoor to outdoor concentrations. In these two studies, similar fungal genera were identified both indoors and outdoors, highlighting the strong influence of regional dust and climatic conditions. Additionally, all investigations emphasized the importance of continuous IAQ monitoring and ventilation management, particularly in high-occupancy or healthcare environments.

One of these studies investigated fungal contamination inside and outside the Holy Mosque in Mecca, KSA. Similar fungal genera were detected indoors and outdoors. Additionally, fungal concentration indoors ranged from 1.1 × 10^2^ to 1.72 × 10^5^ CFU/m^3^, while outdoor concentrations reached 10^6^ CFU/m^3^. The authors reported that the source of this high quantity may be the elevated levels of suspended dust emitted into the air from constructions nearby. Continuous assessing of microbial air contamination in such buildings is highly recommended as millions of visitors are present in the area particularly in Ramadan and Hajj seasons (Mashat, 2015). Two other studies were held in educational buildings (classrooms, offices, food courts, toilets, etc.) in Qatar (Rub et al., 2021) and KSA (Najjar, 2025). In the first study, airborne samples were collected from three schools by filtration followed by cultural analysis for fungi and bacteria in addition to 16S rRNA PCR analysis of bacterial taxa (Rub et al., 2021). Notably, the highest indoor airborne fungal concentration was 51 CFU/m^3^, very low concentration compared to that of bacteria (971 CFU/m^3^). The study reported highest bioaerosol concentrations in food court and recommended the control of air conditioning filters. After the filters were changed, bioaerosols’ concentration dropped implying that the outdoors may be the main source of these microbes. The second study was performed in a University in KSA, samples were collected by impaction, followed by culture and ITS rDNA analyses (Najjar, 2025). Here, the total fungal concentration was 971 CFU/m^3^, where *Aspergillus* genus dominated among others (298 CFU/m^3^). Quantification revealed highest concentrations in classrooms (400 CFU/m^3^) followed by staff offices (239 CFU/m^3^), toilets (171 CFU/m^3^), and labs (161 CFU/m^3^). The authors reported the importance of continuous monitoring of IAQ, considering fungi as indicators of IAQ as well as looking into possible sources of contamination in their future investigations. Seasonal airborne fungal contamination in a nineteen-floor offices building was analyzed in Kuwait (Almatawah et al., 2022). Airborne samples were collected by impaction at two different levels of ventilation shafts (S1: Basement to floor 10 & S2: 9th to 19th floor) and analyzed by culture. Levels of fungal contamination was highest in the summer (25,288 ± 10,763 CFU/m^3^ at S1 & 10,168 ± 1,033 CFU/m^3^ at S2), followed by winter, and was negligible in the spring and autumn seasons at both levels. Notably, concentrations were higher at S1 and higher indoors than outdoors (1,470 ± 1,295 CFU/m^3^ in the winter & 53 ± 18 CFU/m^3^ in the summer). The difference in contamination levels between indoors and outdoors in both seasons is related to the outdoor meteorological data that are not favorable for the growth of fungal genera identified. Two investigations were held in hospitals in Kuwait (Habibi et al., 2022; Asadzadeh et al., 2025). Asadzadeh et al. investigated distribution of fungal contamination indoors and outdoors and performed antifungal susceptibility testing and molecular fingerprinting of environmental and clinically relevant isolates (Asadzadeh et al., 2025). Airborne sampling was realized, as well as swab sampling for AC ducts and surfaces in several wards of a hospital. The samples were analyzed by culture and DNA analysis by sequencing (ITS rDNA, or partial sequencing of *β*-tubulin using ITS1, ITS4 and other primer pairs). Fungal taxa identified indoors were similar to those identified outdoors. These aspergilli and environmental molds are a major cause of seasonal allergies and opportunistic infections in vulnerable individuals. The second study investigated airborne fungal taxa indoors during the COVID-19 pandemic in two hospitals dealing with COVID-19 patients (Habibi et al., 2022). Airborne samples were collected by filtration in hospital laboratories, pharmacies, near the main entrance, etc. The sequencing of 18S rRNA targeting ITS1 and ITS2 was performed. The main fungi identified were *Aspergillus* (*A. ruber*)*, Penicillium* (*P. desertosrum*)*, Fusarium* (*F. proliferatum*) *and Saccharomyces* (*S. cerevisiae*) and all can cause diseases to humans, animals, and plants. According to the study, routine monitoring of indoor bioaerosols is crucial because it helps anticipate potential pathogenic, infectious, or other effects on human health.

### 4.2 Fungal contamination on surfaces

Only one study investigated fungal contaminations on surfaces specifically on mosque carpets (Alsaif et al., 2020) in KSA to assess the prevalence of infectious fungal species. Samples were collected from 100 Mosques in Riyadh by swab sampling and were subsequently cultured on Sabouraud dextrose agar. High prevalence of fungal organisms including *Aspergillus, Alternaria, Bipolaris, Candida, Cladosporium, Curvularia, Rhodotorula*, and other molds and yeasts was reported, suggesting the need to implement new strategies and laws to improve hygiene awareness level among worshipers and caretakers, aiming to limit the spread of foot fungi.

### 4.3 Fungal contamination in dust

Regarding fungal contamination in dust samples, a study tested the efficiency of different filter types in trapping microorganisms through collecting dust samples from AC filters and analyzing, by culture, bacterial and fungal microorganisms (Al-Abdalall and Al-Abkari, 2017). It was reported that dust samples from buildings with good conditions were the most contaminated by microorganisms. Additionally, the most efficient filters were “complex filters” while “sponge filters” were the least efficient. Another study aimed to investigate different allergenic components originating from indoors sources (Hasnain et al., 2017). Dust samples were collected from vacuuming carpets in different rooms of houses. Culture of collected samples yielded the identification of different allergenic fungal taxa such as *Aspergillus, Penicillium, Cladosporium, Alternaria, Fusarium, Rhizopus and Mucor.* A third study investigated indoor fungal contamination from dust samples also collected from AC filters in residential, agricultural, and industrial zones (AlMulla et al., 2022). Culture of the collected samples revealed identification of different fungal taxa in the three study zones as well as different levels of contamination. The highest contamination level was in Industrial zones (4,000 CFU/100 mg) followed by agricultural zones (953 CFU/100 mg) then residential zones (76 CFU/100 mg). The study reported the reliability of assessing IAQ through AC filter dust sampling and recommended continuous monitoring and additional investigations into the indoor and outdoor environments of these respective zones.

## 5 Discussion: consequences on health and recommendations

People spend most of their time indoors, especially in the GCC countries during the extremely hot and humid seasons, thus they are more exposed to indoor airborne microbial contaminants. Several studies previously cited have used cascade impactors to analyze and separate the breathable and inhalable airborne particles (Abdelrahman et al., 2022). According to their sizes, these particles could be inhaled and penetrate various levels of human’s respiratory system and could potentially cause serious health problems. Following WHO’s updated guidelines in 2024, pathogen-laden particles expelled from respiratory track are considered as infectious respiratory particles (IRPs). WHO highlighted that IRPs span a continuous spectrum of sizes and recommends avoiding rigid cut-off points separating ‘droplets’ from ‘aerosols’, focusing instead on exposure context such as distance, ventilation, duration and the multi-pathway nature of transmission (World Health Organization, 2024).

Although investigations on bacterial and fungal contaminations in the indoor environment were limited in the selected geographical area, some of the microbial species reported in the reviewed studies have been linked in the broader international literature to negative health outcomes. Exposure to airborne microbes could have various effects on human health depending on their allergenic, infectious, and pathogenic effects (Haleem Khan and Mohan Karuppayil, 2012; Annesi-Maesano et al., 2013; Chawla et al., 2023) as well as the exposure period (Fakunle et al., 2022), health condition of the person (Kumar et al., 2022; Walser et al., 2015), and microbial concentration in the inhaled air (Kumar et al., 2022). Studies comparing indoors to outdoor levels of bacteria and fungi often reported a ratio of indoor/outdoor microbes greater than one (Habibi et al., 2023; Abdelrahman et al., 2022; Almatawah et al., 2022; Asadzadeh et al., 2025) significantly influencing their environmental bioburden. Such microbial species require serious management and close monitoring to limit their risks on human health as much as possible (Zhang and Srinivasan, 2020). These health implications should be seen as risk-based conclusions rather than concrete proof of illness burden in GCC populations due to the lack of region-specific epidemiological evidence.

In a health-care setting, hospital-acquired illnesses, occupancy levels, and an array of IAQ metrics, including temperature, relative humidity, and carbon dioxide (CO_2_), have all been linked to airborne microbes (Hiwar et al., 2021; Hassan and Zeeshan, 2022). Although hospitals should theoretically have a perfect IAQ, numerous studies showed that such buildings have several bacterial and fungal species disseminated in the air, leading to indoor health challenges and adverse patient prognosis (Baudet et al., 2021; Brambilla and Capolongo, 2019). It has been demonstrated that surfaces, air, and indoor structures, including ventilation systems, serve as pathogen reservoirs. In some situations, these germs – and more dangerously the drug-resistant ones – can survive for several months in a hospital setting (La Fauci et al., 2017; Kramer et al., 2006). Pathogens and opportunistic pathogens can thrive, proliferate rapidly, and infect new individuals, especially those with a compromised immune system (Corse et al., 2020; Smith et al., 2018).

In workplaces, residential and educational settings, where individuals are exposed for longer periods of time to bioaerosols and settled dust, elevated fungal and bacterial loads were linked to inflammatory-related disorders including asthma, allergic rhinitis, and respiratory discomfort (Corse et al., 2020; Spilak et al., 2015; Hamilos, 2010). Dust from AC filters and carpets have been found to harbor high concentrations of pathogenic bacteria and fungi that can be inhaled or transferred to skin, thus highlighting the importance of regular cleaning and disinfection to limit exposure.

Moreover, the studies performed in Kuwait highlighted the increase of airborne bacterial and fungal loads, which is probably due to a higher humidity during this season offering a more suitable environment for microbial growth (Habibi et al., 2023; Almatawah et al., 2022). Comparable results from other warm regions across the globe have reported similar indoor microbial dynamics in humid seasons (Sadigh et al., 2021; Andualem et al., 2019; Akhtar et al., 2025).

Besides, reporting contextual factors of sampling sites was limited. Occupancy density such as number of occupants, duration of stay, activity levels, cleaning procedures, such as ventilation conditions during sampling, maintenance and cleaning history of HVAC were not clearly presented. Such factors would strongly influence indoor microbial loads, leading to biased comparisons for spatial and seasonal variations. It is therefore recommended that future investigations document and publish standardized information on building operation conditions in accordance with international sampling strategy principles and structured monitoring checklists. Such harmonization would enhance cross-study comparability and support evidence-based mitigation strategies.

Additionally, species identification was missed or incomplete in different studies where identification only covered genus level. For better knowledge of exposure to these bioaerosols on human health, it is recommended to identify the species level. This would help understand risk level as some fungi are directly pathogenic (Al Hallak et al., 2023; Walsh et al., 2004; Thirugnanasambandam et al., 2024; Brown, 2023; Al-Humiany, 2010), and/or indirectly toxic if they produce allergenic/toxic compounds (Alam et al., 2022; Bensassi et al., 2015; Aleksic et al., 2017; Charpin-Kadouch et al., 2006; Middeleer et al., 2019). The most common microorganisms in an environment can be identified by using the new generation sequencing (NGS) techniques to access the full panel of microorganisms present. An environment’s aerosol composition can be further established by use of NGS techniques to identify numerous microorganisms, including fastidious organisms that are not easily cultured (Hou et al., 2023). However, such methods do not provide an efficient quantification of the microbes present in a given environment and would need to be complemented with other methods to assess microbial concentrations (e.g., RT-PCR or CFU). Therefore, a complete analysis of indoor air contaminants should preferably include methods yielding both quantitative and qualitative results.

From the reported studies, several species related to *Aspergillus* genus were identified (*A. niger, flavus, fumigatus, ruber, ustus*) (Habibi et al., 2022; Najjar, 2025; Hasnain et al., 2017). Each of these species may impact differently humans. Although it is not considered directly pathogenic, *A. niger* produces carcinogenic mycotoxins, such as ochratoxin A, Fumonisin B2&B4 (Fog Nielsen, 2003; Frisvad et al., 2011). *A. fumigatus* is a pathogenic fungus reported to cause different pulmonary infections and produce various immune-suppressive mycotoxins (Latgé, 2003; Kwon-Chung and Sugui, 2013). Also, several bacterial genera (*Bacillus*, *Staphylococcus*, etc.) have been shown to actively contribute to infections and allergic sensitivities (Rasli et al., 2021). Details on possible consequences on human health of airborne bioparticles are widely available in the literature (Pringle, 2013).

Moreover, dose–response studies indicate that increased exposure raises the likelihood of asthma flare-ups (Pringle, 2013). Therefore, quantification of identified species plays a key role in classifying the level of contamination in a specific indoor environment. The higher the concentration of bioaerosol, the more the chance for it to be inhaled. Although local guidelines regarding concentrations of airborne aerosols relation to IAQ contamination level are not yet underlined, international ones may serve as guides. International guidelines on IAQ have been underlined by different authorities including the World Health Organization (WHO), the French National Agency for Environmental and Occupational Health safety (ANSES), Occupational Safety and Health- Administration (OSHA) of United States, etc. (World Health Organization, 2009; World Health Organization. Regional Office for Europe, 2010; HAL INRAE, 2016; Rapport de l’Anses d’expertise collective, 2013). To improve consistency and allow better comparison between studies, commonly used sampling and analytical methods can be aligned with the ISO 16000 indoor air standard series. For example, ISO 16000-16 describes long-term sampling of airborne molds using filtration (International Organization for Standardization, 2008), ISO 16000-18 specifies short-term sampling by impaction onto agar media, etc. (International Organization for Standardization, 2011). Inclusion of standardized sampling and analysis methods in such investigations imply coherences with international guidelines, allow better understanding of methodology and more accuracy in results. Knowing the importance of investigations on indoor microbial contaminants, and identification of taxa to the species level, quantification of identified taxa would provide clearer vision and clarification of risk of contamination. As per the international guidelines of different health authorities, contamination level is considered low with a concentration <500 CFU/m^3^, hazardous with a concentration range 500-1000 CFU/m^3^ and extremely hazardous above 1,000 CFU/m^3^ (World Health Organization, 2009; Rapport de l’Anses d’expertise collective, 2013). General trends from the available studies indicate that indoor bacterial concentrations in GCC buildings often range from 100 to 500 CFU/m^3^, predominantly including *Staphylococcus, Acinetobacter, Bacillus,* and *Pseudomonas*. For the fungal concentrations, the concentrations were variable depending on the building type analyzed, ranging from 51 to 170,000 CFU/m^3^, with *Aspergillus, Penicillium, Cladosporium, Alternaria, and Fusarium* mostly identified. Seasonal fluctuations (increased counts during colder periods) and more importantly the existence of drug-resistant organisms indicate that indoor microbiological monitoring ought to complement current chemical pollutant assessments for the protection of public health. Particularly MRSA which is not only restricted to healthcare facilities, creates an important threat, and overlooking its detrimental effects, would establish a dangerous trend for healthcare systems and the overall well-being of the community (Al-Saleh et al., 2022; Shaari and Bakar, 2023).

In conclusion, the studies reported here show that indoor airborne bacteria and fungi are at the origin of infections, allergic reactions, respiratory irritation, and even long-term harmful effects are present in high concentrations indoors in the GCC region. This review highlights the crucial need for proper ventilation, filtration, and hygiene in all built environments. Given the unique climatic conditions of the GCC, including frequent dust storms carrying potential pathogens along with environmental microbes and seasonal high particulate loads, region-adapted measures such as enhanced HVAC maintenance schedules, and highly efficient filtration standards may be particularly relevant. Therefore, more effective risk mitigation in these situations may be supported by regionally calibrated microbiological reference levels and adaptive building-management techniques. Indeed, no single intervention is enough to control indoor microbial contamination. Effective improvement involves a coordinated set of actions combining moisture management, filtration optimization, HVAC hygiene and maintenance, cleaning practices monitoring, and occupancy/operational control. Standardized guidelines for a close microbiological monitoring of indoor environments for the area need to be optimized. These should take into consideration several factors such as the extreme weather conditions pushing people to spend more time indoors, the climatic pressures and human based activity, namely the extra usage of antibiotics creating more multi-drug-resistant organisms. Such actions would have a significant impact on the overall public health in these countries.
